# Clinical and genetic evaluation of children with short stature of unknown origin

**DOI:** 10.1186/s12920-023-01626-4

**Published:** 2023-08-21

**Authors:** Qianqian Zhao, Yanying Li, Qian Shao, Chuanpeng Zhang, Shuang Kou, Wanling Yang, Mei Zhang, Bo Ban

**Affiliations:** 1 School of Medicine, Qingdao University, Qingdao, Shandong 266071 P.R. China; 2 Department of Endocrinology, Genetics and Metabolism, Affiliated Hospital of Jining Medical University, Jining Medical University, 89 Guhuai Road, Jining, Shandong 272029 P.R. China; 3 Chinese Research Center for Behavior Medicine in Growth and Development, 89 Guhuai Road, Jining, Shandong 272029 P.R. China; 4 Medical Research Center, Affiliated Hospital of Jining Medical University, 89 Guhuai Road, Jining, Shandong 272029 P.R. China; 5 Department of Paediatrics and Adolescent Medicine, The University of Hong Kong, 21 Sassoon Road, Pokfulam, Hong Kong, 999077 P.R. China

**Keywords:** Short stature, Whole-exome sequencing, Genetic defects, Mutation, Clinical phenotypes

## Abstract

**Background:**

Short stature is a common human trait. More severe and/or associated short stature is usually part of the presentation of a syndrome and may be a monogenic disease. The present study aimed to identify the genetic etiology of children with short stature of unknown origin.

**Methods:**

A total of 232 children with short stature of unknown origin from March 2013 to May 2020 were enrolled in this study. Whole exome sequencing (WES) was performed for the enrolled patients to determine the underlying genetic etiology.

**Results:**

We identified pathogenic or likely pathogenic genetic variants in 18 (7.8%) patients. All of these variants were located in genes known to be associated with growth disorders. Five of the genes are associated with paracrine signaling or cartilage extracellular matrix in the growth plate, including *NPR2* (*N* = 1), *ACAN* (*N* = 1), *CASR* (*N* = 1), *COMP* (*N* = 1) and *FBN1* (*N* = 1). Two of the genes are involved in the RAS/MAPK pathway, namely, *PTPN11* (*N* = 6) and *NF1* (*N* = 1). Two genes are associated with the abnormal growth hormone-insulin-like growth factor 1 (GH-IGF1) axis, including *GH1* (*N* = 1) and *IGF1R* (*N* = 1). Two mutations are located in *PROKR2*, which is associated with gonadotropin-releasing hormone deficiency. Mutations were found in the remaining two patients in genes with miscellaneous mechanisms: *ANKRD11* (*N* = 1) and *ARID1A* (*N* = 1).

**Conclusions:**

The present study identified pathogenic or likely pathogenic genetic variants in eighteen of the 232 patients (7.8%) with short stature of unknown origin. Our findings suggest that in the absence of prominent malformation, genetic defects in hormones, paracrine factors, and matrix molecules may be the causal factors for this group of patients. Early genetic testing is necessary for accurate diagnosis and precision treatment.

## Background

Short stature is one of the most common reasons for attendance at pediatric endocrinology clinics, and it is defined as a height that is more than 2 standard deviations (SDS) below the mean for the same age, sex and population [1]. Linear growth in childhood is a process regulated and influenced by many factors involving many genes and influenced by genetics, hormones, nutrition and the environment [2, 3]. These factors influence the proliferation and differentiation of cells and/or the development and growth of bones, which are the main determinants of height. It is widely accepted that height is strongly regulated by genetic factors. Genetic factors account for 80% of human height outcomes and are suspected to be the main cause of individual growth differences [4]. Therefore, traditional assessments based on clinical findings, supplemented by laboratory and imaging studies, have not been able to determine the cause of growth disorders in these children.

The etiology of short stature may vary depending on the degree and type of growth retardation, alongside other accompanying clinical indications. In cases where a genetic mutation is suspected to be responsible for an individual’s short stature, it is recommended to use genetic testing as a means of obtaining an accurate diagnosis. This approach typically allows clinicians to identify any relevant genetic factors that may contribute to the individual’s condition. However, most patients with short stature are nonsyndromic, and the clinical symptoms associated with short stature are diverse and vary with age. Some of these patients showed a mild phenotype of well-characterized monogenic disorders, such as Noonan syndrome [5] and Leri-Weill dyschondrosteosis (*SHOX* gene defect) [6]. Another group of patients had alleles in genes that led to nonspecific phenotypes, such as those with heterozygous pathogenic alleles in the *ACAN* [7], *NPR2* [8], *NPPC* [9], and *IHH* [10] genes. This becomes a real challenge when these patients have no clear phenotype and no obvious signs or symptoms. Alternatively, these studies may support the hypothesis that rare allelic variants, many of which are unique to single families, may have a greater influence on height determination and lead to short stature without significant effects from other systems. Thus, although the diagnostic role of clinical genetic testing for short stature has been established, the genes and rare variant alleles that contribute to short stature in patient populations have not been fully elucidated.

Whole exome sequencing (WES) has been successfully used to discover gene variations as monogenic causes of growth disorders. Approximately 25–40% of short stature children with previously unknown etiology can receive a molecular diagnosis using appropriate genetic testing techniques [3]. After appropriate modern genetic diagnostic procedures, these patients should be excluded from the diagnosis of short stature of unknown origin. This extreme heterogeneity in a population of unexplained short stature inevitably affects the choice of therapy with recombinant human growth hormone (rhGH). In addition, molecular diagnosis is important because it may guide treatment decisions, facilitate tailored management, and provide adequate genetic counseling. Therefore, the purpose of this study is to apply the WES method to evaluate the genetic etiology of growth disorders in children with short stature of unknown origin and to describe their clinical characteristics and response to rhGH therapy.

## Methods

### Subjects

**Fig. 1:**
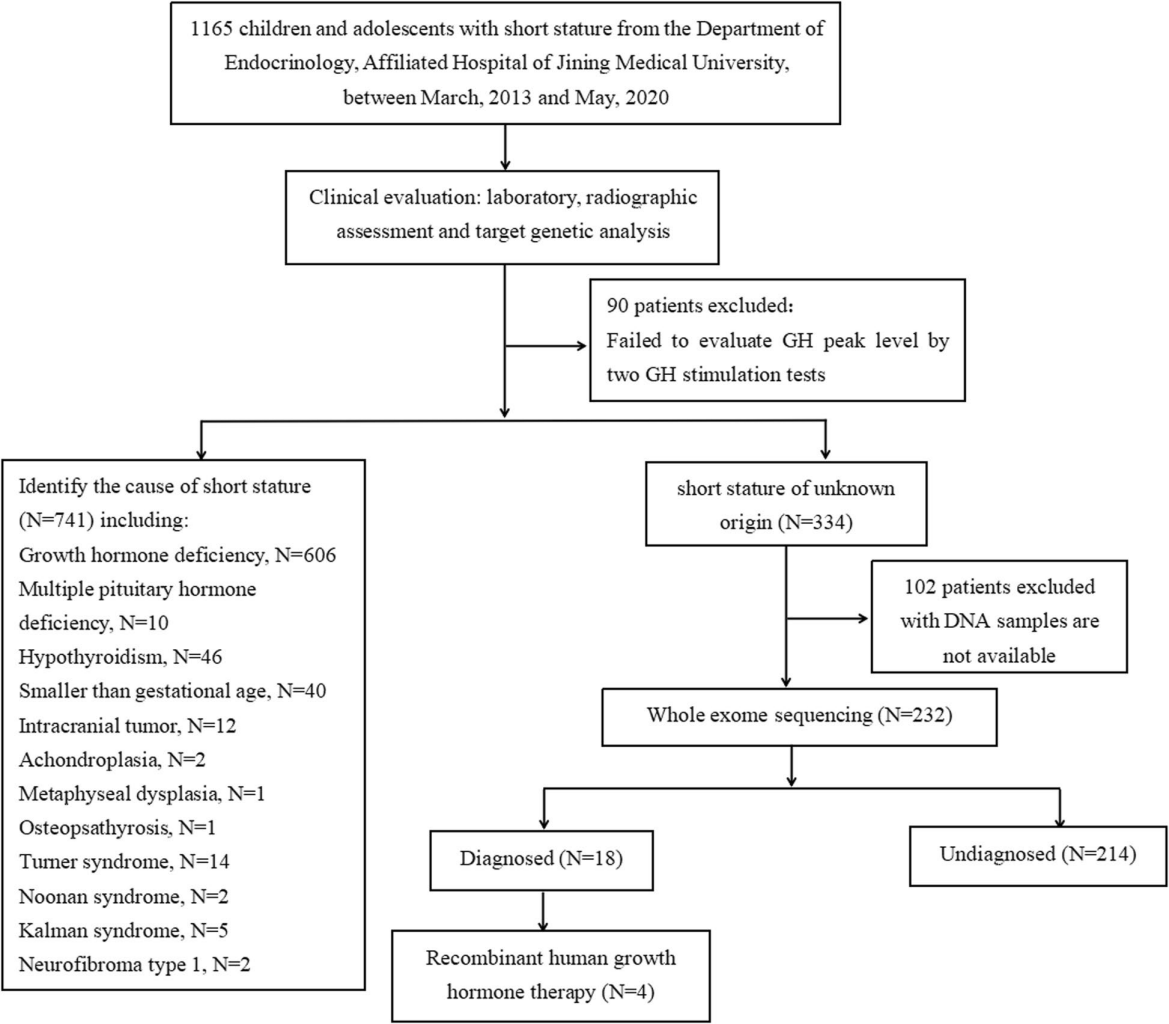
Flowchart of study subjects

**Fig. 2:**
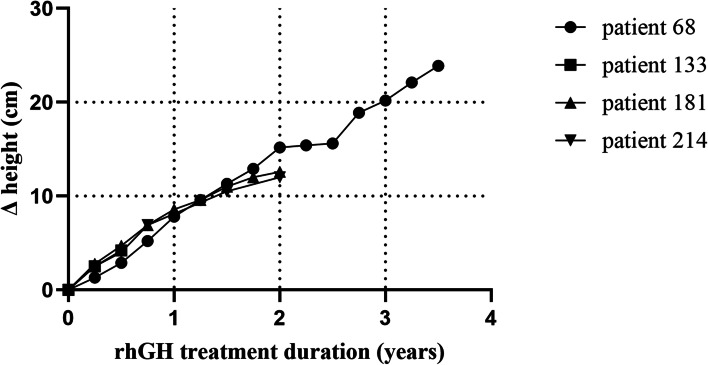
Change in height of the study population after rhGH treatment. rhGH: recombinant human growth hormone

**Fig. 3:**
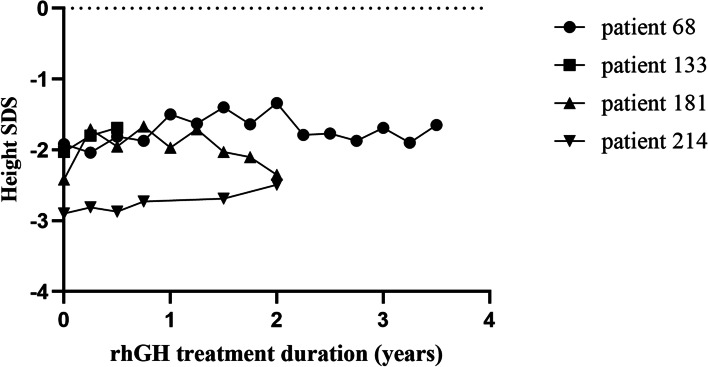
Height SDS of the study population after rhGH treatment. Height SDS: height standard deviation scores; rhGH: recombinant human growth hormone

The subjects of this study were children with short stature of unknown origin who visited the Department of Endocrinology, Genetics and Metabolism, Affiliated Hospital of Jining Medical University from March 2013 to May 2020. The hospital is a comprehensive tertiary hospital with multiple disciplines, and all patients were evaluated by geneticists. Of the 1,165 children and adolescents with short stature, 90 patients were not evaluated by the two growth hormone stimulation tests and were excluded, the cause of short stature could be identified in 741 patients and there was no clear origin in 334 subjects. Among the 334 subjects with unknown origin of short stature, 102 DNA samples were not available, and thus, 232 short stature children and adolescents were finally included in the study. The participant selection process is shown as a flow chart in Fig. 1. The 232 children and adolescents with short stature were unrelated individuals. In total, there were 154 males and 78 females, and the average age was 10.4 ± 3.6 years. All subjects were selected based on the following inclusion criteria: the height of the participants was more than two SDS below the average height for the same race, age and sex; an appropriate birth weight for gestational age; and adequacy of GH was confirmed by at least two GH stimulation tests with concentrations > 10 ng/mL. We excluded patients with preterm birth, small for gestational age, and significant signs of malformation, including severe malformation, microcephaly, neurodevelopmental delay, intellectual disability, or skeletal dysplasia [11]. We did not exclude patients with minor malformations, defined as unusual morphological features found in the general population that have no serious medical or cosmetic significance for the affected individual [12]. In addition, participants with identified causes of short stature or chromosomal abnormalities or those for which DNA samples were not available were excluded from this study Figs. 2 and 3.

### Clinical evaluation

Clinical evaluations included anthropomorphic measurements, laboratory examinations and imaging evaluations. The height and weight of the anthropometric index were measured by a stadiometer (accuracy of 0.1 cm) and an electronic scale (accuracy of 0.1 kg). Height SDS was calculated according to the normal range of Chinese children [13]. Body mass index (BMI) was calculated as weight in kilograms divided by the square of height in meters and expressed as SDS based on the growth curve of Chinese children as a reference [14]. Puberty was assessed by physical examination based on Tanner stages. Participants with testicular volume less than 4 ml and no pubic hair in boys and no breasts and no pubic hair in girls were considered prepubertal [15]. Two GH stimulation tests (levodopa stimulation test and insulin hypoglycemia stimulation test) were performed to determine GH peaks. The serum GH concentration was determined by chemiluminescence (Access 2, Beckman Coulter, USA) with a sensitivity of 0.010 µg/L. Serum IGF-1 and insulin-like growth factor binding protein 3 (IGFBP-3) levels were measured by the chemiluminescence immunometric method (DPC IMMULITE 1000 analyzer, SIEMENS, Germany) with intra- and interassay CVs for IGF-1 of 3.0% and 6.2%, respectively, and intra- and interassay CVs for IGFBP-3 of 4.4% and 6.6%, respectively. IGF-1 SDS were calculated based on IGF-1 levels matched to healthy children and adolescents of the same age and sex [16]. Left hand and wrist X-ray bone age (BA) determination (Ysio SIEMENS, Germany) was conducted using the Greulich and Pyle method [17].

### Genetic examination

Genomic DNA was extracted from peripheral blood using the Blood DNA Midi Kit (D3494-04, Omega Bio-Tek, GA, USA). WES was performed using an Agilent SureSelect V6 capture kit for enrichment of coding exonic sequences (Agilent Technologies, CA, USA), and the indexed DNA fragments were sequenced by BGISEQ-500 (BGI, Shenzhen, China) to achieve 100× average coverage with paired-end sequencing.

After the sequencing data were filtered and quality controlled, all clean reads were aligned to the GRCh37/hg19 assembly of the human genome with Burrows‒Wheeler Aligner software (BWA, version 0.7.15). Variant calling included single nucleotide polymorphisms (SNPs) and small insertions and deletions (InDels), which were detected using HaplotypeCaller of GATK (version 3.7). The resulting data (in variant call format –VCF) were annotated with ANNOVAR. The median average of the target bases was 147x, with 99% of the target bases having ≥ 10x coverage.

### Evaluation of the genetic results

Based on data from the 1000 Genomes Project (http://www.ncbi.nlm.nih.gov/variation/tools/1000 genomes), Genome Aggregation Database (gnomAD: http://gnomad.broadinstitute.org/) and Exome Aggregation Consortium (http://exac.broadinstitute.org), rare nonsynonymous variants were selected, and common polymorphisms with a minor allele frequency (MAF) of 0.1% or above in the general population were excluded. The functional consequence of each variant was predicted by several in silico programs (SIFT, PolyPhen-2, Mutation Taster, LRT Prediction, CADD and M-CAP). Sanger sequencing was performed to validate the candidate variant identified. The pathogenicity of the variants identified during genetic analysis was classified according to the American College of Medical Genetics and Genomics/Association for Molecular Pathology (ACMG/AMP) guidelines [18].

### Statistical analysis

Continuous variables are presented as the mean ± standard deviation or median (interquartile range). Categorical variables are displayed as numbers and percentages. To compare differences between two groups, Student’s t-test was used for normally distributed variables, the Kruskal-Wallis test was used for nonnormally distributed variables, and the chi-square test were used for categorical variables. Statistical analysis was performed with R 4.0.2 (https://www.R-project.org).

## Results

### The patient cohort

The clinical characteristics of this cohort are described in Table 1. WES was performed for a total of 232 children with short stature of unknown origin, of which of 154 (66.38%) were male and 78 (33.62%) were female. The average age and bone age of the selected participants were 10.4 ± 3.6 years and 8.8 ± 4.1 years, respectively. The mean height SDS of patients was − 2.76 ± 0.73. The height SDS of the patients with the gene variant was significantly lower than that of the patients without the gene variant (*P* = 0.023). All individuals showed sufficient GH, and the peak GH was 14.99 ± 4.50 ng/mL. The median IGF-1 SDS and IGFBP-3 levels were − 0.68 (-1.53-0.34) and 4.61 (3.66–5.63) µg/mL, respectively. The IGF-1 SDS of patients carrying the genetic variant was significantly lower than that of patients without the genetic variant (*P* = 0.038). Therefore, genetic testing is more suitable for patients with severe short stature and lower levels of IGF-1.

**Table 1:** Clinical characteristics of the study population

Characteristic	All	Without genetic variant	With genetic variant	P
Number	232	214	18	
Sex (male %)	154 (66.38%)	141 (65.89%)	13 (72.22%)	0.585
Age (years)	10.4 ± 3.6	10.5 ± 3.5	9.0 ± 3.8	0.101
Bone age (years)	8.8 ± 4.1	8.9 ± 4.1	7.5 ± 4.5	0.221
Height (cm)	126.73 ± 19.23	127.69 ± 18.78	116.50 ± 21.40	0.021
Height SDS	-2.76 ± 0.73	-2.62 ± 0.69	-3.08 ± 1.04	0.023
Body weight (kg)	27.21 ± 10.61	27.59 ± 10.65	23.15 ± 9.49	0.056
BMI (kg/m^2^)	16.21 ± 2.27	16.20 ± 2.30	16.38 ± 1.99	0.266
BMI SDS	-0.53 (-1.14-0.03)	-0.60 ± 0.91	-0.19 ± 1.30	0.156
Sit height/height	0.55 ± 0.05	0.55 ± 0.04	0.57 ± 0.08	0.200
Arm span/height	0.97 ± 0.04	0.97 ± 0.03	0.97 ± 0.07	0.407
IGF-1 (ng/mL)	183.00 (101.00-325.00)	193.00 (105.50–331.00)	96.00 (74.12–158.50)	0.006
IGF-1 SDS	-0.68 (-1.53-0.34)	-0.62 (-1.49-0.39)	-1.31 (-1.81–0.59)	0.038
IGFBP-3(ug/mL)	4.61 (3.66–5.63)	4.76 ± 1.31	3.82 ± 1.03	0.003
Father’s height (cm)	167.21 ± 5.18	155.46 ± 5.95	153.76 ± 8.34	0.530
Mother’s height (cm)	155.32 ± 6.19	167.25 ± 5.15	166.80 ± 5.64	0.599
Peak GH (ng/mL)	14.99 ± 4.50	14.96 ± 5.58	15.35 ± 4.66	0.340
Pubertal stage				0.494
In prepuberty (n, %)	137 (59.05%)	125 (58.41%)	12 (66.67%)	
In puberty (n, %)	95 (40.95%)	89 (41.59%)	6 (33.33%)	

*Height SDS* height standard deviation scores, *BMI *body mass index, *BMI SDS *body mass index standard deviation scores, *IGF-1* insulin like growth factor-1, *IGF-1 SDS *insulin like growth factor-1 standard deviation scores, *IGFBP-3 *insulin-like growth factor-binding protein-3, *GH *growth hormone

### Genetic variants identified

For 7.8% of children in this cohort (18/232), pathogenic or likely pathogenic variants with potential clinical significance in genes with a known impact on growth were identified. Of these variants, seven were classified as pathogenic, and 11 were classified as likely pathogenic. All these variants were identified in genes already associated with growth disorders (Table 2): *PROKR2* (*N* = 2), *PTPN11* (*N* = 6), *ANKRD11* (*N* = 1), *NPR2* (*N* = 1), *NF1* (*N* = 1), *ACAN* (*N* = 1), *GH1* (*N* = 1), *FBN1* (*N* = 1), *COMP* (*N* = 1), *IGF1R* (*N* = 1), *ARID1A* (*N* = 1), and *CASR* (*N* = 1). Only one of these mutations was a frameshift mutation. All other mutations were missense and predicted to be pathogenic by a variety of silico tools.

**Table 2:** Pathogenic and likely pathogenic variants identified by whole exome sequencing of the study population

ID	Gene	Transcript Variant	Protein Variant	Functional annotation	Mutation Status	Evidence for ACMG/ AMP classification	ACMG/AMP classification
56	*PTPN11*	NM_002834.3 c.1507G > C	p.Gly503Arg	Missense	Het	PS1 + PS4 + PM1 + PM2 + PP2 + PP3	P
68	*PTPN11*	NM_002834.3 c.317 A > G	p.Asp106Gly	Missense	Het	PS4 + PM1 + PM2 + PM5 + PP2 + PP3	P
77	*ANKRD11*	NM_001256182.1,c.2579 C > T	p.Ser860Leu	Missense	Het	PS1 + PM1 + PM2 + PP3	LP
79	*PTPN11*	NM_002834.3 c.922 A > G	p.Asn308Asp	Missense	Het	PS1PS3PM1PM2PP2PP3	P
87	*NPR2*	NM_003995.3 c.2629_2630delAG	p.Ser877fs*10	Frameshift	Het	PVS1 + PM2	LP
88	*IGF1R*	NM_000875.4 c.847G > A	p.Ala283Thr	Missense	Het	PM1 + PM2 + PP1 + PP3	LP
91	*PTPN11*	NM_002834.3 c.922 A > G	p.Asn308Asp	Missense	Het	PS1 + PS3 + PM1 + PM2 + PP2 + PP3	P
108	*NF1*	NM_001042492.2, c.3104T > C	p.Met1035Thr	Missense	Het	PS1 + PM1 + PM2	LP
110	*PROKR2*	NM_144773.3 c.1057 C > T	p.Arg353Cys	Missense	Het	PS1 + PM2 + PM5	LP
119	*ACAN*	NM_013227.3, c.2367delC	p.Ser790fs*20	Frameshift	Het	PVS1 + PS1 + PM2	P
123	*GH1*	NM_000515.4, c.125G > A	p.Arg42His	Missense	Het	PM1 + PM2 + PM3 + PP3	LP
133	*FBN1*	NM_000138.4, c.4152G > A	p.Met1384Ile	Missense	Het	PM1 + PM2 + PM5 + PP2	LP
144	*COMP*	NM_000095.2, c.949G > T	p.Asp317Tyr	Missense	Het	PM1 + PM2 + PM5 + PP3	LP
181	*ARID1A*	NM_006015.4, c.6234G > C	p.Glu2078Asp	Missense	Het	PM1 + PM2 + PM5	LP
182	*CASR*	NM_001178065.1,c.2068 C > T	p.Arg690Cys	Missense	Het	PS1 + PM1 + PM2 + PP3	LP
202	*PTPN11*	NM_002834.3 c.236 A > G	p.Gln79Arg	Missense	Het	PS1 + PM1 + PM2 + PP2 + PP3	P
214	*PTPN11*	NM_002834.3 c.922 A > G	p.Asn308Asp	Missense	Het	PS1 + PS3 + PM1 + PM2 + PP2 + PP3	P
232	*PROKR2*	NM_144773.3, c.685G > C	p.Gly229Arg	Missense	Het	PS1 + PM1 + PM2	LP

*Het* heterozygous, *AD *autosomal dominance, *LP *likely pathogenic, *P *pathogenic

### Clinical phenotypes

The phenotypic characteristics of the patients carrying a pathogenic or likely pathogenic mutation are shown in Table 3. In total, eighteen pathogenic or likely pathogenic variants from unrelated individuals were identified. Their height SDS ranged from − 2.03 SD to -3.94 SD. Nine of the eighteen patients had familial short stature (FSS), with the height of a father or mother below 160 cm or 150 cm, respectively. However, blood samples were obtained from the parents of only 5 patients. Parental testing using Sanger sequencing revealed that for 3 patients, the variants were inherited from their affected mother. For 1 patient, the variant was inherited from his affected father, and for 1 patient, the variant was a de novo mutation (Table 3). Additionally, one patient had a slightly advanced bone age at prepubertal age (chronological age was 3 years old and bone age was 4 years old for a patient with *ACAN*), whereas the majority of the patients had a markedly delayed bone age.

**Table 3:** Clinical characteristics at the first evaluation of the patients with pathogenic or likely pathogenic variants identified by whole exome sequencing

ID	Gene	Sex	Age (years)	BA (years)	Height SDS	BMI SDS	SH/H	AS/H	GH peak (ng/mL)	Birth weight (kg)	Birth length (cm)	IGF-1 (SDS)	IGFBP3 (mg/L)	Duration of GH treatment (years)	∆Height SDS	Father’s height (cm)	Mother’s height (cm)	Inherited	Additional phenotypic features
*56*	*PTPN11* (p.Gly503Arg)	F	6	3	-3.64	-0.09	0.58	0.95	20.12	2.9	50	-1.16	3.21	-	-	160	142	-	High arched palate, low posterior hairline
*68*	*PTPN11* (p.Asp106Gly)	M	10	7.5	-2.29	-0.27	0.55	0.99	10.40	3.3	50	-2.13	3.88	3.5	0.27	160	147	-	High arched palate, premature atrial
*77*	*ANKRD11* (p.Tyr761fs)	F	14	12	-3.31	-0.51	0.54	0.99	18.88	3.2	50	-0.43	6.77	-	-	175	150	maternal	Congenital heart defects, delayed bone age
*79*	*PTPN11* (p.Asn308Asp)	M	14	10.5	-2.79	-0.38	0.52	0.95	14.87	3.9	51	-1.53	4.27	-	-	174	149	de novo	Congenital heart defects, cryptorchidism
*87*	*NPR2* (p.Ser877fs*10)	M	5	2	-2.38	-0.36	0.56	0.94	10.31	3.1	50	-1.30	3.10	-	-	168	149	maternal	-
*88*	*IGF1R* (p.Ala283Thr)	M	15	13	-4.69	0.21	0.55	0.95	17.37	3	50	0.57	5.83	-	-	160	160	paternal	High arched palate
*91*	*PTPN11* (p.Asn308Asp)	M	10	6	-3.06	-0.29	0.54	0.96	11.79	2.7	50	-1.81	3.35	-	-	164	155	-	Congenital heart disease, pulmonary valve stenosis, atrial septal defect
*108*	*NF1* (p.Met1035Thr)	M	4	2	-2.41	2.27	0.58	0.98	12.43	3	50	-0.59	4.37	-		165	165	-	15 milk coffee spots with a diameter greater than 5 mm
*110*	*PROKR2* (p.Arg353Cys)	M	14	12.5	-2.25	-1.33	0.50	0.98	14.29	3.4	50	-0.13	-	-	-	174	165	-	Delayed development of secondary sexual characteristics
*119*	*ACAN* (p.Ser790fs*20)	F	3	4	-2.24	2.14	0.59	0.94	15.38	3.4	50	-1.81	3.00	-	-	175	161	-	Slightly advanced bone age
*123*	*GH1* (p.Arg42His)	M	5	3	-2.91	-0.59	0.87	0.96	24.23	2.6	50	-1.30	2.77	-	-	163	149	-	Low levels of insulin-like growth factor 1
*133*	*FBN1* (p.Met1384Ile)	M	10	6	-2.03	-0.92	0.53	1.01	10.97	3.4	50	-0.58	4.09	0.5	0.34	159	153	-	Viral encephalitis, sinus arrhythmia, high arched palate
*144*	*COMP* (p.Asp317Tyr)	M	3	-	-3.05	1.77	0.61	0.92	24.63	3.1	52	-0.76	2.74	-	-	169	132	maternal	Limited knee mobility, Lumbar spine tilted forward significantly, Forearm flexion, Metaphyseal dysplasia
*181*	*ARID1A* (p.Glu2078Asp)	F	12	12	-2.42	-0.64	0.53	0.99	11.70	2.8	55	1.28	6.42	2	0.07	170	149	-	Cystic enlargement of both ovaries
*182*	*CASR* (p.Arg690Cys)	M	8	5	-3.67	-0.79	0.56	0.90	22.52	2.7	50	-1.63	3.22	-	-	170	146	-	Hypothyroidism, low blood calcium level
*202*	*PTPN11* (p.Gln79Arg)	M	15	13	-3.94	-0.97	0.56	0.97	18.05	3.1	50	-1.93	3.81	-	-	159	160	-	Cubitus valgus, perpetual left superior vena cava, cryptorchidism
*214*	*PTPN11* (p.Asn308Asp)	M	4	2	-2.90	-0.37	0.53	0.92	11.02	3.4	50	-2.08	-	2	0.21	170	160	-	Cryptorchidism
*232*	*PROKR2* (p.Gly229Arg)	F	5	2.5	-2.26	0.99	0.57	0.98	15.84	3.8	52	-0.96	3.72	-	-	177	162	-	Delayed bone age

*M* male, *F *female, *BA *bone age, *SH/H *sit height/height, *AS/H *arm span/height

Five patients had rare genetic variants in genes (*NPR2, ACAN, CASR, COMP*, and *FBN1*) directly involved in paracrine signaling or the cartilage extracellular matrix in the growth plate. Patient #133 carried a heterozygous *FBN1* mutation and presented with severely delayed bone age (BA-CA: -4 years) and additional phenotypic features, including viral encephalitis, sinus arrhythmia, and high arched palate. Patient #144 carried a heterozygous mutation in *COMP* (c.949G > T) and presented with short stature accompanied by skeletal dysplasia. His height SDS was − 3.05 SD. The height of his mother was 132 cm, and she presented with additional phenotypic features, such as limited knee mobility, lumbar spine tilted forward significantly, forearm flexion and metaphyseal dysplasia. These patients showed only a mild clinical phenotype and were within the normal range of body proportions, including the sit height/height and arm span/height (Table 3). The bone age x-ray evaluation for these patients showed no abnormalities, except for minor metaphyseal chondrodysplasia in patient #144, and further lumbar spine radiographs showed unnatural morphology, which indicated minor dysplasia.

Two patients, including one boy and one girl, carried the *PROKR2* gene variant. The 14-year-old boy exhibited delayed development of secondary sex characteristics, a mid-parental height (MPH) of 176 cm, and a bone age delay of 1.5 years. The 5-years-old girl was still being followed up. She presented a MPH of 163 cm, and exhibited a significant delay in bone age (BA-CA: -2.5 years).

Seven patients had pathogenic variants in two genes involved in the RAS-MAPK pathway: *PTPN11* (*N* = 6) and *NF1* (*N* = 1). At the first evaluation, their clinical presentation was not sufficient to allow the diagnosis of Noonan syndrome (OMIM #163,950) or neurofibromatosis type 1 (OMIM #162,200). Patient #108 carried a heterozygous *NF1* mutation and presented with 15 milk coffee spots with a diameter greater than 5 mm on the chest and underarms. His head circumference was in the normal range of 48.5 cm, and his academic performance was acceptable. However, he is now only 4 years old and needs follow-up monitoring. Patient #56, patient #68, patient #79, patient #91, patient #202 and patient #214 all carried mutations in *PTPN11*; these patients demonstrated different specific facial features, congenital heart anomalies or cryptorchidism.

Patient #77 carried a mutation in the *ANKRD11* gene, which encodes an ankyrin repeat domain-containing protein, corresponding to the phenotype of KBG syndrome (OMIM#148,050) with an autosomal dominant inheritance pattern. Her height SDS was − 3.31 SD, BMI SDS was − 0.51 SD, peak GH concentration was 18.88 ng/mL, IGF-1 SDS showed a low level of -0.43 SD, and additional phenotypic characteristics included congenital heart disease and delayed bone age.

Patient #87 and patient #123 carried *IGF1R* and *GH1* gene mutations, respectively; these genes are related to the abnormal growth hormone/insulin-like growth factor 1 (GH/IGF-1) axis. Patient #87 showed adequate IGF-1 and GH levels. The IGF-1 SDS was 0.57, and peak GH was 17.37 ng/mL. Short stature may be associated with IGF-1 insensitivity, and the clinical phenotype was high arched palate. Patient #123 carried the *GH1* gene variant. This patient had sufficient GH with a peak GH of 24.23 ng/mL. The level of IGF-1 was low, and IGF-1 SDS was − 1.30. Partial GH insensitivity may be present in this patient.

### Growth response to rhGH treatment

Of the 18 patients with a pathogenic or likely pathogenic variant, 4 received rhGH for 0.5 to 3.5 years (Figs. 2 and 3). The age range of these patients at rhGH treatment ranged from 4 to 12 years, and these patients showed varying degrees of catch-up growth during treatment with rhGH (Δ height SDS range: 0.07 SD to 0.34 SD). Among these four patients, one patient was treated for half a year, one patient was treated for 3.5 years, and two patients were treated for 2 years. Patient #133 carried the *FBN1* gene variation, and after 0.5 years of treatment with rhGH, the SDS of height improved by 0.34 SD. rhGH treatment had been used for only 0.5 years in this patient; thus, conclusions about the efficacy of rhGH treatment could not be reached. Patient #68 and Patient #214 both carried the *PTPN11* gene variant, and the rhGH treatment duration was 3.5 years and 2 years, respectively. For these patients, the height SDS only improved by 0.27 SD and 0.21 SD, respectively. In addition, during 2 years of rhGH treatment, there was no significant improvement in the height SDS (Δ height SDS: 0.07 SD) of patient #181, who carried the *ARID1A* variant.

## Discussion

The present study evaluated the complex genetic etiology of short stature in a population of unrelated individuals using the WES method. From our cohort of 232 children with short stature of unknown origin at the time of diagnosis, 18 pathogenic or likely pathogenic mutations that were associated with growth disorders were identified. These genetic variants affect diverse pathways involved in growth, including the endocrine system, paracrine growth factors, extracellular matrix, and intracellular signaling molecules in the growth plate. Patients with different genetic variants showed significant differences in response to rhGH treatment. Early genetic diagnosis may lead to better clinical management and treatment choices.

Many children with short stature of unknown origin show mild clinical features that can be observed in some syndromes. However, these features are not sufficient to diagnose a specific syndrome. Therefore, some special syndromes with a wide range of clinical phenotypes, especially for patients with mild clinical features, may be misdiagnosed as idiopathic short stature. The final diagnosis may not be clear until molecular genetic and/or biochemical markers are identified. Recognizing the etiology of short stature and elucidating the gene-specific response to rhGH therapy is one of the major challenges facing pediatric endocrinology. Pathogenic or likely pathogenic variants were revealed in 7.8% of children with short stature in our study (18/232). This finding is similar to that of our previous study in which WES was performed for 93 patients with growth hormone deficiency (GHD); this study yielded a molecular diagnostic rate of 7.5% [19]. A recent review mentioned that genetic testing for short stature children of unknown etiology yielded a diagnosis rate of between 4% and 16%, which increased with the severity of short stature and the fact that the child had additional characteristics, such as deformations or intellectual disabilities [20].

The genetic variants we identified in this study were all heterozygous variants with an autosomal dominant pattern of inheritance. This finding is similar to that of a previously reported study by Ahn J et al., in which 14 of 144 patients with short stature were identified as carrying pathogenic or likely pathogenic variants, and all patients showed a dominant inheritance pattern [21]. In addition, autosomal recessive genetic disorders are also causes of short stature and may account for a relatively small proportion of short stature patients, especially for those with nonsyndromic short stature. A recent study of the genetics of short stature in China showed that the genetic diagnosis rate of patients with idiopathic short stature was 11.3%. Of these patients, 72% carried monoallelic variants consistent with the underlying autosomal dominant (AD) condition or disease feature, and 10% carried biallelic variants consistent with Mendelian expectations for an autosomal recessive (AR) condition [22]. In addition, in the above study, dual diagnosis of short stature was present in 0.5% of patients with syndromic short stature, while no dual diagnosis was found in patients with idiopathic short stature [22]. The lack of identification of genetic causes of short stature due to autosomal recessive disorders and dual diagnosis in the present study may be related to regional differences and the inclusion criteria for the patient population. In our previous genetic testing of GHD patients, no dual diagnosis or autosomal recessive genetic disease of short stature was identified [19].

The genetic causes of linear growth disorders identified in previous reports are highly heterogeneous, affecting a variety of cellular pathways. Furthermore, any regulatory mechanism that alters growth plate chondrogenesis may be a genetic cause of growth disorders [23]. A notable aspect of this study was the frequency of heterozygous mutations detected in genes previously associated with skeletal dysplasia (*NPR2, ACAN, CASR, COMP*, and *FBN1*), confirming the dose effect of cartilage matrix proteins in growth and development. In patients with short stature disorder of unknown origin in the present study, these gene variants showed a mild clinical phenotype, causing difficulties for accurate diagnosis. Among these gene variants, *NPR2* and *ACAN* are more common in patients initially diagnosed with idiopathic short stature [24, 25]. In the present study, one case of a *NPR2* gene variant and one case of a *ACAN* gene variant were identified. Patient #87 carried heterozygous frameshift mutations of *NPR2* (c.2629_2630delAG); however, no significant skeletal phenotype was observed. Previous studies have shown that heterozygous mutations in *NPR2* appear to be associated with mild and variable growth disorders without a distinct skeletal phenotype compared with homozygous or compound heterozygous mutations in *NPR2* that cause severe short stature and body disproportion [26]. Patient #119 carried a heterozygous frameshift mutation in the *ACAN* gene, and the clinical characteristics showed slightly advanced bone age. A recent study noted that 47.4% of patients with *ACAN* variants in the Chinese population showed advanced bone age [27]. Functional aggregative proteoglycan haploid deficiency leads to premature maturation of hypertrophic chondrocytes and premature invasion of vascular cells and osteoblasts in the growth plate, which has been suggested as a potential mechanism for advanced bone age, premature growth cessation, and early epiphyseal fusion in patients with *ACAN* mutations [28, 29].

In this study, we separately identified two patients with rare variants p.Gly229Arg and p.Arg353Cys in *PROKR2* who showed delayed development of secondary sexual characteristics. In addition, we also identified four patients with a common *PROKR2* (p.Trp178Ser) variant. This variation was classified as pathogenic, including in other previous studies involving short stature [21]. However, we did not include the p.Trp178Ser in *PROKR2* as a possible cause of short stature, since it has been described in several others clinical exomes with no mention to this phenotype. *PROKR2* is traditionally known as a gene implicated in hypogonadism hypogonadotropic, involved in pituitary ontogenesis [30]. Additionally, some investigators have also suggested that the *PROKR2* is associated with hypothalamic pituitary development and that children carrying this variant may present with short stature [31]. In addition, a *PROKR2* variant was identified in patients born small for gestational age, without any facial deformity, with not catching up on growth [32]. In a previous study, an idiopathic short stature patient with a *PROKR2* missense mutation was identified [21].

In our study, we also identified seven patients with pathogenic or likely pathogenic variants affecting the RAS-MAPK pathway. Six of the patients carried missense mutations in the *PTPN11* gene, which is the most commonly mutated gene associated with Noonan syndrome (OMIM #163,950). Noonan syndrome is usually diagnosed by specific clinical criteria [33]. Facial features and/or typical heart malformations often raise doubts about the diagnosis. However, the phenotype of the patients analyzed in this study was mild, with only 2 patients having congenital heart disease, which is often associated with the clinical phenotype of Noonan syndrome. The characteristic facial appearance of Noonan syndrome varies somewhat across ages. However, with increasing age, the facial features of affected children become increasingly atypical. Mutations in the *PTPN11* gene cause large clinical phenotypic variability, so milder mutations in the *PTPN11* gene may result in less severe cases that are insufficient to meet the clinical diagnostic criteria for Noonan syndrome. If a patient has a mild or atypical presentation, genotyping could establish the diagnosis [33]. In addition, a previous study revealed atypical faces of Chinese Noonan syndrome patients and the lack of genetics training in China, which may have led to missed diagnoses in the past without next-generation sequencing [34].

Although most short stature patients can receive rhGH treatment to improve adult height, not all short stature patients are eligible or suitable for rhGH treatment. Clear molecular diagnosis of patients is crucial for deciding to continue/stop growth hormone therapy [35]. Individuals with growth hormone receptor (GHR) deficiency have a poor response to rhGH treatment. The short stature caused by abnormal growth plate-related genes makes it difficult to achieve a good therapeutic effect with rhGH treatment. For Bloom syndrome, rhGH treatment is contraindicated. For children who can be treated with rhGH, identifying genetic mutations can help determine the therapeutic effect of rhGH. If children homozygous for *GH1* gene deletions are treated with rhGH, growth hormone antibodies are easily produced, leading to poor efficacy. After rhGH treatment, the levels of IGF-1 in children with IGFR deficiency can be significantly increased, but sensitivity decreases [31]. In this study, 4 out of 18 patients with pathogenic gene variants received rhGH treatment. However, one of the patients had only been using rhGH for 0.5 years. Thus, there is not enough evidence to draw any conclusions about the efficacy of the treatment. Two patients with Noonan syndrome were treated for 2 and 3.5 years, and only limited growth catch-up was observed. Several previous studies have reported a correlation between rhGH treatment response and genotype in Noonan syndrome, with patients carrying the *PTPN11* pathogenic variant having a poor response to rhGH treatment [36, 37]. However, rhGH treatment has been reported to improve the height and growth rate in patients with Noonan syndrome, but there was no significant difference in rhGH treatment response in Noonan syndrome patients carrying the *PTPN11* gene variant compared with those not carrying the *PTPN11* gene variant [38]. In addition, a study by E-M Seok et al. showed that in rhGH-treated Noonan syndrome patients, the improvement in growth rate was significantly higher in patients carrying the PTPN11 gene variant than in those not carrying the PTPN11 gene variant [39]. This suggests that the response to rhGH treatment in Noonan syndrome patients carrying the *PTPN11* gene variant is currently controversial. Further confirmation is needed with large samples and long-term follow-up cohort studies. However, most of the current research evidence supports a reduced rhGH treatment response in patients carrying the *PTPN11* pathogenic gene variant [40]. The mechanism by which the *PTPN11* gene variant leads to a reduced rhGH treatment response has not been elucidated but may be due to the role of SHP-2 as a negative regulator of GH receptor signaling [41, 42].

Several potential limitations of the present study should also be considered. Parental samples were not available for all patients; thus, some patients lacked trio testing, which should be performed in the future to improve the diagnostic rate. In addition, functional studies of pathogenic variants have not been conducted and can be added in future studies. Finally, in addition to the variants reported in this study, we cannot confirm the presence of other candidate variants in any patient. Even though we conducted WES for each patient, it is possible that some rare or private variants were missed due to the current limitations of sequencing technology. Furthermore, the interpretation of genetic variants is an ongoing and complex process, and new tools and information may become available in the future that can reclassify variants previously considered benign or of uncertain significance.

## Conclusion

We performed genetic analysis of WES in Chinese children with short stature of unknown origin. Pathogenic or likely pathogenic genetic variants were identified in 18 of the 232 patients (7.8%). Genetic testing may help determine the underlying cause of short stature, helping to make an accurate diagnosis and treatment decisions. Even in the absence of malformation features, short stature may be caused by genetic defects in hormones, paracrine factors, matrix molecules, intracellular pathways, and fundamental cellular processes. Further studies should be conducted to test gene-specific GH reactivity as a necessary first step toward better personalization of short stature management.

## Data Availability

The data that support the findings of this study are not publicly available due to restrictions by General Data Protection Regulation (GDPR), but are available from the corresponding author on reasonable request.

## Abbreviations

WES: Whole exome sequencing
GH/IGF-1: Growth hormone/insulin-like growth factor-1
rhGH: Recombinant human growth hormone
IGFBP-3: Insulin-like growth factor binding protein 3
BA: Bone age
SNPs: Single nucleotide polymorphisms
InDels: Insertions and deletions
FSS: Familial short stature
MPH: Mid-parental height
NF1: Type 1 neurofibromatosis
